# Beam model development and clinical experience with RadCalc for treatment plan quality assurance in online adaptive workflow with an MR‐linac

**DOI:** 10.1002/acm2.70125

**Published:** 2025-05-19

**Authors:** Urszula Jelen, Zoë Moutrie, Jack D Aylward, Michael G Jameson

**Affiliations:** ^1^ GenesisCare Alexandria New South Wales Australia; ^2^ South Western Sydney Cancer Services New South Wales Health Sydney New South Wales Australia; ^3^ University of New South Wales Sydney New South Wales Australia; ^4^ Ingham Institute for Applied Medical Research Liverpool New South Wales Australia; ^5^ Medical Physics School of Applied Sciences University of the West of England Bristol UK; ^6^ Medical Physics University Hospitals Bristol and Weston NHS Foundation Trust Bristol UK; ^7^ University of Wollongong Wollongong New South Wales Australia

**Keywords:** adaptive radiotherapy, independent MU verification, MR‐linac, patient specific quality assurance, RadCalc

## Abstract

**Purpose:**

The aim of this work was to report on the optimization, commissioning, and validation of a beam model using a commercial independent dose verification software RadCalc version 7.2 (Lifeline Software Inc, Tyler, TX, USA), along with 4 years of experience employing RadCalc for offline and online monitor unit (MU) verification on the Elekta Unity MR‐linac (MRL) for a range of clinical sites.

**Methods:**

Calculation settings and model parameters, including the Clarkson integration settings and radiation/light field offset, have been systematically examined and optimized, and pitfalls in the use of density inhomogeneity corrections and in off‐axis calculations were investigated and addressed. The resulting model was commissioned by comparing RadCalc calculations to measurements for a variety of cases, selected following relevant recommendations, ranging from simple fields in a water tank to end‐to‐end point dose measurements in an anthropomorphic phantom.

**Results:**

For simple geometries, the agreement was within 2%, and for complex geometries, within 5%. When validating against the Monaco (Elekta AB, Stockholm, Sweden) treatment planning system (TPS), for 39 clinical commissioning plans, the mean total point dose difference was −0.3 ± 0.8% (−2.0%–1.1%). Finally, when applied retrospectively to 4085 clinical plan calculations, the agreement with the TPS was 0.3 ± 1.1% (−4.8%–4.2%), with fail rates of 0.1% for total point dose (discrepancy > 4%) and 0.3% for individual fields (discrepancy > 10%).

**Conclusions:**

Improved calculation agreement with the TPS and therefore increased confidence in the online QA, opened the way for an automated and physics‐light independent MU verification workflow within our MRL program.

## 1 INTRODUCTION

The pursuit of the highest precision in therapeutic dose delivery has prompted the development of MRI‐linacs (MRLs), integrated systems combining linear accelerators and magnetic resonance (MR) scanners,^1^, ^2^ which through reliable soft tissue delineation, enable daily adaptation of treatment to inter‐fraction changes in the patient's anatomy.

In the MRL treatment workflow, a new plan is generated at each fraction. Quality assurance (QA) for such online‐adapted plans relies solely on independent monitor unit (MU) verification, as dose measurement is possible only after the treatment has been delivered. This requires both efficiency and calculation accuracy. Minimizing the time spent on QA reduces the patient burden, particularly given the inherently extended duration of online adaptive treatments, while accurate calculations reduce the incidence of out‐of‐tolerance results, which otherwise necessitate time‐critical investigations and decision making while the patient remains on the treatment couch.

One challenge in implementing independent MU verification is modeling of the magnetic field effects on dose deposition, which, while addressed in the treatment planning systems (TPSs) dedicated for use with MRLs, is still not widely available in stand‐alone MU verification software, often relying on less sophisticated calculation algorithms.

RadCalc (Lifeline Software Inc., Tyler, TX, USA) is one of the first and few MU verification software tools capable of modeling magnetic field effects on dose distribution. However, early study demonstrating its application for high‐field MRL^3^ utilized an older software version, which did not account for these effects. The commissioning of RadCalc version 7.1.4, which introduced magnetic field modeling,^4^ has been demonstrated by Price et al.,^5^ albeit for the low‐field MRIdian (ViewRay, USA) system, where these effects are less pronounced. Sung et al.^6^ investigated RadCalc 7.1.4 for a high‐field system, however, focusing primarily on the impact of beam modeling data on the calculation accuracy. Published work for high‐field MRLs, addressing model configuration, calculation settings, and algorithm limitations, as well as long‐term clinical use, remains limited.

At our institution, expanding on the work by Graves et al.,^3^ RadCalc has been used for independent verification of the Monaco (Elekta AB, Stockholm, Sweden) TPS MU calculation for both reference (offline) and adapted (online) plans for the Unity MRL (Elekta AB, Stockholm, Sweden) since 2020. Building on this experience, the aim of this work was threefold:

1. to report on the investigations of parameters, calculation settings, and challenges within RadCalc arising due to the specifics of the Unity MRL, highlighting previously unreported limitations,
2. to present the optimization, commissioning, and validation of the resulting beam model,
3. and to demonstrate its real‐world implementation, showcasing performance beyond commissioning tests, discussing the impact of software updates and model improvements, and highlighting its role in achieving an automated, physics‐light treatment workflow.

## 2 METHODS

### 2.1 Input data and the initial model

Published RadCalc models for MRLs are based on calculated rather than measured input data,^3^, ^5^, ^6^ due to bore size limitations, which impose depth and field size constraints for the base data acquisition shown to affect the calculation accuracy.^6^ Therefore, upon verification that it closely matches our machine data (pass rate of 100% for all depth dose curves and mean pass rate of 99.1% for profiles at 1%/1 mm criteria, mean output factor difference of 0.4%), the Monte Carlo modeled data, generously provided by the University of Iowa team,^3^ have been implemented in the initial beam model v1, created in RadCalc 6.4 available at the time of our MRL commissioning.

### 2.2 Software upgrades and initial model revisions

Since the initial commissioning, we have undergone three RadCalc software upgrades requiring model re‐validation and, in some instances, parameter re‐tuning. After upgrading to RadCalc 7.1.4, the radiation/light field offset (RLO), previously used as a workaround to achieve better agreement in absence of profile asymmetry modeling,^3^ was restored to its intended purpose of accounting for the rounded leaf tips in the model version v2. More recently, plan re‐calculations conducted in preparation for the upgrade to RadCalc 7.2 revealed an increased discrepancy between RadCalc and the TPS for simple irregular fields (on average by 1%) and for clinical plans (on average by 0.6%). This could be attributed to the change in the field perimeter and scatter factor calculation, disclosed in the software change log, and prompted RLO tuning, described in “Radiation/light field offset” of Section 2.3.2, for the model version v3 and thereafter.

Furthermore, a recent retrospective review of our clinical MU verification results revealed a higher incidence of total point dose fails (i.e., exceeding 4% tolerance) for liver cases (4.3%) compared to an overall fail rate of 0.4%. This correlated with the far‐off‐axis position of the target in these cases, prompting a re‐evaluation of base data used for off‐axis ratios (OARs) calculation, described in “Off‐axis ratios” of Section 2.3.2, for the optimized model version v4.

The commissioning and validation of model v4 are described in the subsequent sections. It should be added that testing demonstrated agreement between RadCalc 7.2 and 7.3 within ± 0.2%, and the model v4 was ported to RadCalc 7.3 without changes.

### 2.3 Model optimization, commissioning, and validation

AAPM TG‐219^7^ recommends commissioning secondary MU verification software following the same procedures as used for primary TPS.^8^, ^9^ Model commissioning and validation have been conducted by comparing RadCalc calculation results to commissioning measurements or TPS calculations.

Commissioning data were acquired, as appropriate to the task, using: Farmer chamber 30013 (PTW, Freiburg, Germany) with the calibration traceable to the primary standards laboratory (ARPANSA, Australia), Semiflex 3D chamber 31021 (PTW, Freiburg, Germany) or microdiamond 60019 (PTW, Freiburg, Germany) in MR‐compatible scanning tank Beamscan MR (PTW, Freiburg, Germany), manual 1D MR‐compatible tank MP1 (PTW, Freiburg, Germany), MR‐compatible ArcCheck‐MR (Sun Nuclear, Melbourne, FL, USA) or Zeus MRGRT phantom (Sun Nuclear, Melbourne, FL, USA). It should be noted that the ArcCheck was docked to the treatment couch using a QA platform, rather than its standard lightweight cradle. For details on patient‐specific QA with ArcCheck on Unity MRL the reader is referred to Strand et al.^10^

All TPS calculations were performed with Monaco v5.51.11 (Elekta AB, Stockholm, Sweden). For the phantom cases, a dose grid of 2 mm and Monte Carlo statistical accuracy of 0.5% per calculation were used, and for the patient datasets, clinical settings were employed (2 mm/1%, except for 3 mm/1% for pancreas). The modified Clarkson integration (MCI) algorithm^11^, ^12^ was used for all RadCalc calculations.

Several phantom datasets were used for calculations: a 60 × 45 × 23 cm water phantom representing the Beamscan MR tank (referred to hereafter as the water tank phantom), a 20 × 20 × 20 cm water phantom (referred to hereafter as the water phantom), and a cylindrical phantom with radius of 13.3 cm representing the ArcCheck device (referred to hereafter as the cylindrical phantom) with the relative electron density (RED) of 1.18.

#### 2.3.1 Calculation options

##### Clarkson calculation options and volume averaging

Radial and angular sampling for MCI and volume averaging parameters were chosen considering accuracy (higher sampling resolution yields more accurate results, especially for small apertures) and compatibility with dose grid sizes used in the TPS, whilst maintaining acceptable computation times.

##### Density inhomogeneity correction methods

The equivalent path (EP) and equivalent path with field size scaling (EP+FSS) methods were evaluated by comparing RadCalc and the TPS‐calculated point doses for square fields of varying sizes (from 1 × 1 cm^2^ to 22 × 22 cm^2^) and for clinical commissioning plans in the cylindrical phantom as well as for commissioning plans in patient anatomy. The QA platform, MR coils, and couch structures were removed in some tests to isolate their effect on the inhomogeneity corrections.

#### 2.3.2 Parameter optimization

##### MLC and jaw transmission

MLC and jaw transmission parameters were determined by comparing RadCalc and the TPS point dose calculations in a water phantom for a point located under a leaf in a field with all leaves closed for transmission settings of 0.004, 0.005, and 0.006.

##### Radiation/light field offset

The RLO has limited meaning for the Unity system employing radiation field‐based MLC calibration. Furthermore, the Technical Reference Guide recommends fine‐tuning this parameter to match measurements and/or TPS calculations. To optimize the RLO setting, RadCalc and the TPS point doses were compared for 166 individual fields from 19 commissioning plans (10 prostate SBRT and 9 abdominal or pelvic oligometastatic SBRT) calculated in the cylindrical phantom using three RLO settings: 0.0 cm, 0.07 cm (user manual recommended), and 0.1 cm (derived by scaling the recommended value according to the non‐standard source‐to‐isocenter distance of the Unity MRL). The phantom RED was overridden to 1.0 and the QA platform, couch, and MR coil structures were removed, to avoid interference with the inhomogeneity corrections.

##### Off‐axis ratios

Due to the field size limitation in the in‐plane direction, the largest field available on the Unity system is a rectangle, measuring 57.4 × 22 cm^2^, whereas RadCalc requires square field entries to derive OARs. To optimize off‐axis calculation accuracy, three test models with maximum field sizes of 57.4 × 57.4 cm^2^, 40 × 40 cm^2^, and 22 × 22 cm^2^ were investigated by comparing the agreement between the RadCalc and the TPS point doses for a 3 × 3 cm^2^ field at off‐axis distances ranging from −24 cm to +21 cm in the cross‐plane and from −9 to +9 cm in the in‐plane direction, calculated in the water tank phantom.

##### Calibration factor

While the primary role of the calibration factor is to represent the dose delivered under reference conditions, two corrections have been incorporated into this parameter: a correction for systematic difference between dose‐to‐medium (in the TPS) and dose‐to‐water (in RadCalc) calculation and a factor to account for angular beam output dependence resulting from incomplete helium fill and cryostat manufacturing tolerances.^13^ The former, similarly to the approach recommended in AAPM TG‐329,^14^ was established as the ratio of point doses calculated in the TPS using both the dose‐to‐water and dose‐to‐medium options for clinical commissioning plans representing various indications: prostate, prostate bed, abdominal or pelvic oligometastasis, pancreas, liver, head‐and‐neck, and kidney. While it should be underlined that the quantity reported by Monaco, sometimes referred to as dose‐to‐water‐in‐medium, is not identical with the dose‐to‐water used by conventional algorithms,^15^, ^16^ it appears to be reasonably close for the range of electron densities encountered in tissues.^17^

The actual angular beam output dependence cannot be modeled in RadCalc; instead, a representative value derived from the cryostat transmission characterization curve has been implemented.

#### 2.3.3 Commissioning tests

Following relevant recommendations,^7^, ^8^, ^9^ RadCalc point dose calculations were compared to the values measured during commissioning, as detailed in Table 1.

**TABLE 1:** Model commissioning test cases.

Fields compared	Setup	Measurement
Absolute dose for reference field (10 × 10 cm^2^)	Isocenter and dose reference point at 10 cm depth (SSD = 133.5 cm), gantry 0°	Farmer chamber in a manual 1D tank MP1
Square fields of various sizes (1 × 1 cm^2^–40 × 22 cm^2^)	Isocenter and dose reference point at 10 cm depth (SSD = 133.5 cm), gantry 0°	Semiflex 3D chamber or microdiamond in Beamscan tank
Irregularly shaped fields: L‐shapes and T‐shapes in different orientations, U‐shape, diamond, and chair shape		
Rectangular fields 4 × 14 cm^2^ 12 cm off‐axis cross‐plane and 14 × 4 cm^2^ 7 cm off‐axis in‐plane		
Square fields 3 × 3 cm^2^–16×16 cm^2^ (due to the rectangular tank shape)	Isocenter and reference point in 30 cm depth (SSD = 113.5 cm due to the rectangular tank shape), gantry 270°	Microdiamond in Beamscan tank
Commissioning patient plans (10 prostate 60 Gy/20 fractions, 10 prostate SBRT, and 7 abdominal or pelvic oligometastatic SBRT cases)	Clinical isocenter and gantry angles, dose reference point at chamber location	Semiflex 3D chamber in ArcCheck
End‐to‐end plan	Clinical isocenter and gantry angles, dose reference point at chamber location	Semiflex 3D chamber in Zeus MRGRT phantom

Abbreviation: SSD, source‐to‐surface distance.

#### 2.3.4 Validation tests

Agreement of the point dose calculations between RadCalc and the TPS was assessed for a set of validation cases of increasing complexity listed in Table 2.

**TABLE 2:** Model validation test cases.

Fields compared	Setup	Dataset
Reference field (10 × 10 cm^2^)	Isocenter and dose reference point at 10 cm depth (SSD = 133.5 cm), gantry 0°	Water (tank) phantom (RED = 1)
Square fields of various sizes (1 × 1 cm^2^–50 × 22 cm^2^)		
Irregularly shaped fields: L‐shapes and T‐shapes in different orientations, U‐shape, diamond, and chair shape		
Off‐axis fields 1 × 1 cm^2^–10 × 10 cm^2^ at cross‐plane and in‐plane offsets of 5–9 cm		
Square fields of various sizes of only up to 16 × 16 cm^2^ (due to the rectangular tank shape)	Isocenter in 30 cm depth and reference dose point in 5, 10, 15 and 30 cm (SSD = 113.5 cm due to the rectangular tank shape), gantry 270°	
Commissioning patient plans (10 prostate SBRT and 9 abdominal or pelvic oligometastatic SBRT cases)	Clinical isocenter, dose reference point, and gantry angles	Cylindrical phantom (RED = 1)
Commissioning patient plans (10 prostate 60 Gy/20 fractions, 10 prostate SBRT, 9 abdominal or pelvic oligometastatic SBRT, 5 liver SBRT, and 5 pancreas SBRT cases)	Clinical isocenter, dose reference point, and gantry angles	Patient anatomy

Abbreviations: RED, relative electron density; SSD, source‐to‐surface distance.

### 2.4 Model performance in clinical setting

All clinical MU verifications subsequent to the RadCalc upgrade to version 7.2, involving a total of 4085 online and offline treatment plans, have been reassessed using model v4. Additionally, the clinical results obtained for the same plans using model v3 (in RadCalc 7.2) and for 2108 plans verified using model v2 (in RadCalc 7.1) are presented.

### 2.5 Evaluation

For simple fields, the relative point dose difference between RadCalc calculation and the measurement result or the TPS calculation was recorded for each individual field. For clinical plans, the total relative point dose difference between RadCalc calculation and the measurement result or the TPS calculation was recorded. Additionally, for some validation cases, the largest negative (worst −) and the largest positive (worst +) relative dose difference between RadCalc and TPS calculation for individual fields were recorded. For the analysis, the mean ± standard deviation and the range of relevant parameters were reported. Additionally, for clinical plans, the frequencies of total point dose and individual field discrepancies were derived.

For commissioning, tolerances consistent with the AAPM Practice Guideline 5.a^9^ have been employed: 0.5% for absolute dose calibration, 2% for tests with one and 5% with more parameters departing from the reference conditions, 2% for IMRT plans, and 5% for end‐to‐end test.

The results of validation against the TPS were benchmarked against the AAPM TG‐219^7^ consensus action levels for high dose/low gradient points: 5% for individual fields and 3% for composite plans in homogeneous medium, and 7% for individual fields and 5% for composite plans in heterogeneous medium.

## 3 RESULTS

### 3.1 Model optimization, commissioning, and validation

#### 3.1.1 Calculation options

##### Clarkson calculation options and volume averaging settings

Comparison of the parameters used in this work, set to values equal or finer than manual recommendations, with previously published ones, is presented in Table 3. Furthermore, the volume averaging settings were adapted: the default radius was set to 0.05 cm to be increased in increments of 0.05 cm up to a maximum of 0.4 cm if the calculated dose is not within 5%.

**TABLE 3:** Clarkson calculation options used in this work and reported in the literature.

Parameter	RadCalc manual	Graves et al.^3^	Sung et al.^6^	This work
Radial sampling distance (cm)	0.1	0.5	0.1	0.1
Angular sampling increment (°)	5	5	5	2.5
Radius for primary dose (cm)	0.25	0.8	0.5	0.2
Pixel size for intensity map (cm)	0.25	0.5	0.25	0.2

The resulting calculation times varied depending on the number of fields and on the target size (determining the field area) in the clinical plans. For cases with smaller targets, such as oligometastatic plans, calculation times were in the order of 1–2 s, while for cases with larger targets or utilizing more fields, such as prostate with lymph nodes or pancreas plans, the longest calculation times were approximately 10–15 s.

##### Density inhomogeneity correction methods

**FIGURE 1:**
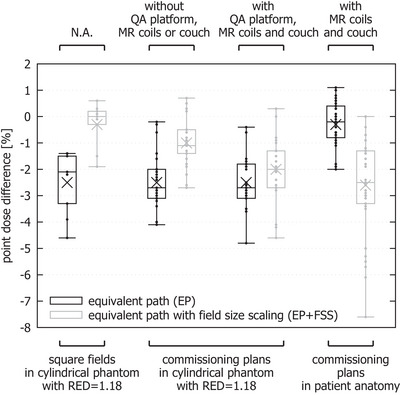
Box plot (cross—mean, crossbar—median, box – 1st and 3rd quartile, whiskers—min/max, dots—all data points) of the point dose differences between RadCalc using different inhomogeneity correction methods and the TPS. RED, relative electron density; TPS, treatment planning system.

The simple EP method led to a notable underestimation of point doses in a cylindrical phantom with RED = 1.18 (Figure 1), suggesting that it is unsuitable in this range of densities. While the EP+FSS method yielded better results for square fields at gantry 0° (mean point dose agreement with the TPS improved by 2.2%) and for clinical plans in absence of QA platform, MR coil and couch (mean agreement improved by 1.5%), it gave mixed results in presence of these contours (mean agreement improved only by 0.5%). Furthermore, the EP+FSS method gave notably worse results for patient cases (mean agreement worsened by 2.3%) (Figure 1).

**FIGURE 2:**
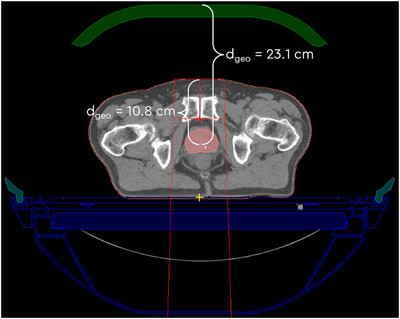
The effect of the MR coil presence in the TPS plan on the field size dependent density correction factor calculation in RadCalc: large ratio between the geometrical depth (23.1 cm) and equivalent depth (11.4 cm) leads to inhomogeneity correction factor = 1.49 while with the coil removed this factor amounts to 0.986. MR, magnetic resonance; TPS, treatment planning system.

This underperformance could be attributed to the mis‐estimation of the inhomogeneity correction factor in the EP+FSS method, caused by the presence of structures representing the MR coils and some of the couch elements, as they may be considered the initial surface for the geometrical depth calculation in the TPS (thinner elements of the couch may be missed by the entrance point search), as shown in Figure 2. For this reason, the EP+FSS method was deemed unsuitable for clinical plan calculations requiring the presence of the anterior MR coil. On the other hand, the simple EP method yielded good agreement in real patient geometries, where densities are closer to the density of water.

#### 3.1.2 Parameter optimizations

##### MLC and jaw transmission

The calculation agreement between RadCalc and TPS for a point located under the leaf for MLC transmission settings of 0.004, 0.005, and 0.006 yielded −18.2%, 1.5%, and 21.5%, respectively. Consequently, the MLC transmission was set to 0.005, with the jaw transmission adjusted to the same value, following the vendor's recommendation.

##### Radiation/light field offset

Comparison of the mean discrepancies between the point doses calculated in RadCalc and the TPS using test models employing RLO settings indicated that an RLO = 0 cm results in the best agreement: 0.2 ± 2.6% (as compared to 0.8 ± 2.7% for RLO = 0.07 cm and 0.9 ± 2.8% for RLO = 0.1 cm).

##### Off‐axis ratios

**FIGURE 3:**
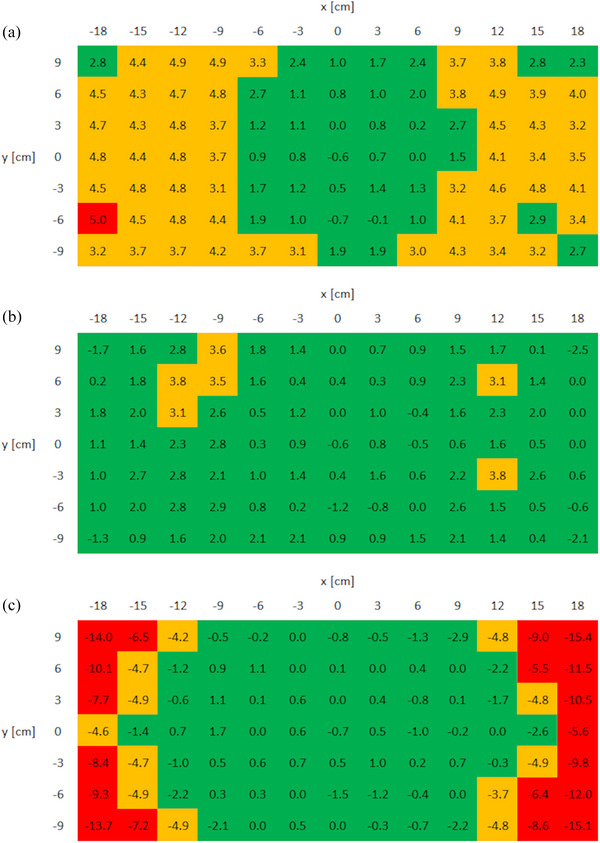
Agreement between the RadCalc and TPS point dose calculation for a 3 × 3 cm field as a function of the field off‐axis position for three RadCalc test models (a) using 57.4 × 57.4 cm^2^, (b) using 40 × 40 cm^2^ and (c) using 22 × 22 cm^2^ field as the largest field for the OAR calculation. OAR, off‐axis ratio; TPS, treatment planning system.

Using the profiles of a 57.4 × 57.4 cm^2^ field for OARs calculation led to an overestimation of the calculated point doses at off‐axis positions of 9 cm and beyond (Figure 3a). While the test model using 22 × 22 cm^2^ field profiles gave the best agreement within ± 12 cm, it led to underestimations further off‐axis (Figure 3c). The best overall agreement was observed using 40 × 40 cm^2^ field profiles (Figure 3b), and this field size was implemented for OAR calculation.

##### Calibration factor

The mean ratio of the point doses calculated in the TPS using the dose‐to‐medium and dose‐to‐water options was 0.995 (kidney 0.989, prostate and head‐and‐neck 0.991, liver 0.992, prostate bed 0.998, and abdominal or pelvic oligometastasis and pancreas 1.003) and was incorporated into the calibration factor. It should be noted that this correction was not applied in calculations in water phantoms.

A value of 0.9922, obtained by averaging the cryostat transmission over the full gantry angle range, was implemented to account for the lower relative output at gantry angles between 42° and 320°, caused by the presence of liquid helium, as opposed to at gantry 0°, where the output is calibrated.^13^ It should be noted that this correction was not used in calculations employing solely the gantry angle of 0°.

#### 3.1.3 Commissioning tests

The calculated absolute dose agreed with the measured dose under reference conditions within 0.1%. The calculated and measured output factors (relative to 10 × 10 cm^2^ in 10 cm depth at isocenter with gantry at 0°) agreed within 1.6% for square fields except for the smallest field (−2.4%), within 1.8% for irregular fields, within 2.1% for rectangular off‐axis fields and within 4.2% for square fields at non‐standard source‐to‐surface distance (SSD) (Table 4). All fields except 1 × 1 cm^2^ were within tolerance.^9^

**TABLE 4:** Comparison of measured and RadCalc calculated output factors for square and irregular fields.

Field	Relative difference (%)
1 × 1^a^	−2.4
2 × 2^a^	0.3
3 × 3	0.3
5 × 5	0.0
15 × 15	−0.5
22 × 22	−0.9
40 × 22	−1.6
U‐shape	1.0
T‐shape (180°)	−0.2
T‐shape (0°)	1.8
T‐shape (90°)	−0.3
T‐shape (270°)	0.6
L‐shape	−0.6
Chair	1.1
Diamond	−0.4
4 × 14 (*x* = −12 cm)	2.1
4 × 14 (CAX)	0.4
4 × 14 (*x* = 12 cm)	1.2
14 × 4 (*y* = 7 cm)	0.6
14 × 4 (CAX)	−0.2
14 × 4 (*y* = −7 cm)	−1.1
3×3 at SSD = 113.5 cm^a^	−1.3
10 × 10 at SSD = 113.5 cm^a^	−2.3
16 × 16 at SSD = 113.5 cm^a^	−4.2

Abbreviations: CAX, central axis; SSD, source‐to‐surface distance.

^a^ measured with microdiamond.

The mean point dose difference between RadCalc and measurements in the ArcCheck was: −2.3 ± 1.0% (−3.7% to −0.3%) for prostate 60 Gy/20 fraction plans, −3.2 ± 0.9% (−4.6% to −2.0%) for prostate SBRT and 0.4 ± 2.0% (−2.8% to 2.9%) for oligometastatic SBRT plans, failing the tolerance in some cases.^9^ The results were slightly improved when enabling FSS, yielding respectively: −1.3 ± 1.0% (−2.5% to 0.9%), −2.4 ± 1.0% (−3.8% to −1.3%) and −0.3 ± 2.0% (−3.0% to 2.8%). For comparison, the mean agreement between the TPS calculations and the measurements for these cases was 0.1 ± 0.1% (−2.3% to 2.3).

Finally, the agreement between RadCalc calculations and point dose measurements for the end‐to‐end plan was within tolerance^9^ −0.3% for the point located in the target and −3.4% for the point placed in the low dose region within an organ‐at‐risk.

#### 3.1.4 Validation tests

**FIGURE 4:**
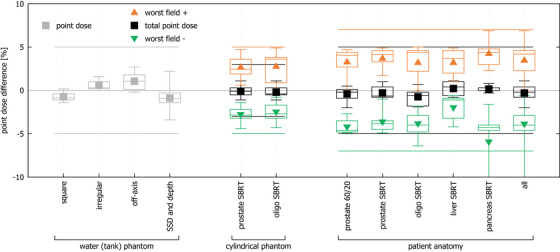
Box plot (symbols—mean, crossbar—median, box – 1st and 3rd quartile, whiskers—min/max,) of point dose differences between RadCalc and the TPS results for all validation cases: simple fields (gray), total point dose differences for composite plans (black), worst field negative differences (green) and worst field positive differences (orange). Lines represent the TG‐219 consensus action levels.^7^ TPS, treatment planning system.

The model validation results are summarized in Figure 4. The mean difference between RadCalc and the TPS was: −0.7 ± 0.5% (−1.4% to 0.2%) for square fields of varying sizes, 0.6 ± 0.6% (0% to 1.6%) for irregular fields, 1.1 ± 0.9% (−0.2% to 2.7%) for off‐axis fields and −0.9 ± 1.0% (−3.4% to 2.2%) for square fields at varying depth and SSDs.

For the clinical prostate and oligometastatic plans calculated on the cylindrical phantom with RED = 1.0, the mean total point difference was: −0.1 ± 0.6% (−1.1% to 1.1%) and −0.2 ± 0.7% (−1.1% to 1.1%). The maximum individual field discrepancies reached 4.7% and 4.9%, respectively.

Finally, for the clinical plans calculated in real patient anatomy (with MR coil and couch structures in place), the mean total point difference was: −0.4 ± 0.7% (−2.0% to 0.5%) for prostate 60 Gy/20 fractions, −0.3 ± 0.7% (−1.0% to 1.0%) for prostate SBRT, −0.7 ± 1.0% (−1.9% to 0.7%) for oligometastatic SBRT, 0.2 ± 0.9% (−0.8% to 1.1%) for liver SBRT and 0.2 ± 0.4% (−0.3% to 0.8%) for pancreas SBRT plans. The respective maximum individual field discrepancies were: −4.9%, ± 4.9%, −6.4%, 4.9% and −15.3%.

In all cases, total point dose differences remained well within TG‐219 consensus action levels^7^ and we adopted a slightly tighter tolerance of 4% for our clinical practice. All except one individual field in a pancreas case also remained within the TG‐219 action levels.^7^ Considering that due to the nature of IMRT delivery, while the point may be in the high dose region of the total plan, it may not be the case for each individual field, for clinical practice we have applied a wider 10% action level, recommended by TG‐219^7^ for points in the gradient area.

### 3.2 Model performance in clinical setting

**FIGURE 5:**
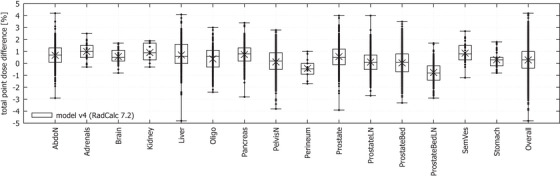
Box plot (cross—mean, crossbar—median, box – 1st and 3rd quartile, whiskers—min/max, dots—all data points) of total point dose differences between RadCalc and the TPS obtained with the optimized beam model v4 applied retrospectively to 4085 plans.

For the 4085 MU verifications performed since RadCalc upgrade to version 7.2, the total point dose agreement between beam model v4 and the TPS was 0.3 ± 1.1% (−4.8% to 4.2%). The results in Figure 5 demonstrate that the mean discrepancies were below 1% for all treatment indications. For comparison, the agreement obtained with model v3 for the same set of plans was 1.1 ± 1.1% (−4.1% to 5.5%) and for 2018 plans verified with model v2 prior to upgrading 0.6 ± 1.5% (−4.3% to 7.7%).

**FIGURE 6:**
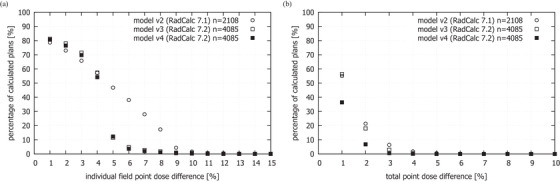
Frequency plots of the individual field and total point dose differences recorded for the model v2 (in RadCalc 7.1), model v3 (in RadCalc 7.2), and for the optimized model v4 in RadCalc 7.2 (*n*—total number of plans).

Frequency plots of the individual field and total point dose differences recorded for all three models are shown in Figure 6. The percentage of total point dose differences exceeding the tolerance of 4% was 1.8% when using model v2 (in RadCalc 7.1), reduced to 0.4% using model v3 (in RadCalc 7.2), and further to 0.1% using model v4 (in RadCalc 7.2). The percentage of point dose differences for individual fields exceeding the tolerance of 10% was, respectively, 1.7%, 0.7%, and 0.3%.

## 4 DISCUSSION

This study presents the first comprehensive evaluation of a secondary MU verification model in RadCalc 7.2 for a high magnetic field MRL. While Sung et al.^6^ in the only previous work applying a RadCalc version capable of modeling magnetic field effects to high‐field MRL, focused on beam data selection and pre‐processing, we describe systematic evaluation of options and parameters affecting the calculation results, highlight and address several limitations and pitfalls and comment on our experience with software upgrades. Furthermore, we present results of rigorous model testing designed following published recommendations^7^, ^8^, ^9^ and, last but not least, we demonstrate performance of our model on a large sample of clinical treatment plans for a range of indications.

### 4.1 Optimization of model parameters and calculation options

The beam model presented in this work resulted from gradual development driven by software updates, as well as clinical performance reviews and evolving clinical requirements.

In order to achieve greater accuracy, we refined the calculation parameters. While the importance of calculation speed for online application should be emphasized, we found that lowering these parameters compared to published works had no significant impact on the online MU verification times, which are largely driven by data transfer. Overall, typical MU verification times are in the order of 2 min.^18^ With the proposed parameters, the calculation itself takes only a few seconds longer, which is outweighed by a reduced incidence of failed results requiring time‐critical investigations in the online scenario.

The field size dependent heterogeneity correction algorithm, while advantageous in simple phantom geometries, led to worse results in the presence of structures representing auxiliary devices: MR coils, couch, and QA platform in the TPS. This could be attributed to the mis‐estimation of the inhomogeneity correction factor demonstrated in “Density inhomogeneity correction methods” of Section 3.1.1 and constitutes a limitation in achievable calculation accuracy, rendering this method unreliable in clinical practice.

A change in the field perimeter calculation introduced in RadCalc 7.2 required re‐visiting the RLO setting described in “Radiation/light field offset” of Section 3.1.2. This underlines the importance of robust post‐upgrade testing, similar to that recommended for primary TPSs.^8^, ^9^

While it might have been preferable to optimize the RLO value using measured patient‐specific QA data as reference, the comparisons with point dose measurements in ArcCheck are hindered by several limitations. Apart from the possible presence of setup uncertainties, the QA platform introduces significant radiological depth variations at oblique beam angles, which limit the accuracy of the equivalent path length‐based calculation. Second, due to the nature of IMRT delivery, it is not always possible to ascertain that the ion chamber remains within low gradient high dose regions for all treatment fields, potentially introducing volume‐averaging effects. Further to this, the multiplug insert used to position ion chamber within the ArcCheck enables only discrete ion chamber positions, which sometimes do not fall within a uniform dose region, especially for small targets. Finally, the ArcCheck device is characterized by a relatively high density, requiring the use of field size scaling for increased inhomogeneity correction accuracy, which, in turn, is impeded by the presence of the couch and QA platform structures. Therefore, RLO optimization was conducted by assessing RadCalc calculation agreement with the TPS in a water‐overridden cylindrical phantom. The additional advantage of this approach is the ability to consider individual field rather than total plan doses, which potentially cancel out some discrepancies. Our results indicate that an RLO value of 0 cm, rather than the 0.07 cm recommended in the manual, grants the best agreement.

Retrospective review of clinical MU verification results revealed a higher incidence of total point dose fails (i.e., exceeding 4% tolerance) in cases with targets positioned farther off‐axis, often beyond limits encountered in the commissioning set. This prompted investigation into the OAR calculation method, modification of base data employed in the model, described in “Off‐axis ratios” of Section 3.1.2, and expansion of our testing suite to include small far‐off‐axis fields. Furthermore, the expanding clinical indication spectrum prompted a refinement of the dose‐to‐water/dose‐to‐medium conversion factor, described in “Calibration factor” of Section 3.1.2. This highlights that initial commissioning and model validation are often aligned with the use intended at the time and that changes in the scope of practice may require their revision.

Finally, we attempted to account for cryostat transmission—a feature for the Unity MRL not addressed in previous works.^3^, ^6^ We have incorporated average cryostat transmission into the model calibration; however, the lack of gantry angle dependent transmission modeling remains a factor contributing to the accuracy limitation. Furthermore, it should be underlined that this parameter requires revisiting following any changes in helium fill level.

### 4.2 Commissioning and validation results

**FIGURE 7:**
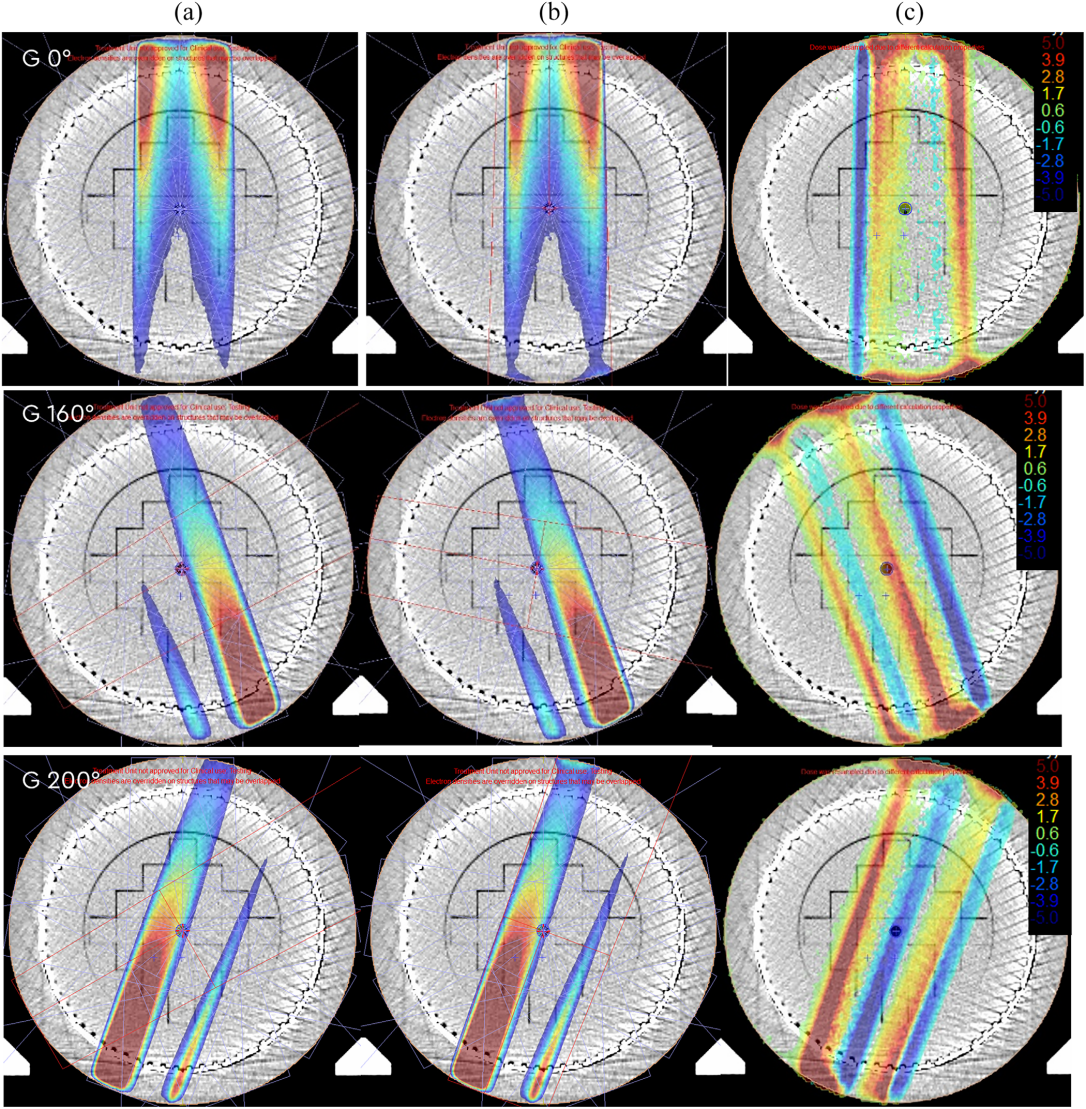
Dose distribution for three typical prostate SBRT fields (at gantry angle 0°, 160°, and 200°) calculated with the Monaco TPS (a) in presence and (b) in absence of the magnetic field and (c) the difference map demonstrating pronounced dose difference at the point of measurement for fields at gantry 160° and 200° due to the profile shifting with depth. TPS, treatment planning system.

Optimized beam model v4 was benchmarked against commissioning measurements, demonstrating agreement for absolute calibration, simple test cases, and end‐to‐end test well within established tolerances,^9^ which, it should be underlined, were designed for the primary TPSs employing more advanced calculation algorithms. However, in some cases, the tolerance was exceeded for clinical commissioning plans delivered to the ArcCheck device. Good agreement observed between these measurements and the primary TPS calculation suggests that shortcomings of the RadCalc calculation, rather than the measurement uncertainties, might be the cause of these discrepancies. Several factors might have contributed to this. As mentioned, the high‐density ArcCheck device requires field size scaling for accurate inhomogeneity correction. However, although the anterior MR coil is not present in this setup, posterior and oblique fields might be affected by the presence of the treatment couch and posterior coil and by the radiological depth variations introduced by the QA platform, impeding the efficiency of the EP+FSS method and leading to a systematic dose underestimation. Another factor, potentially adding to the observed discrepancy, is the lack of modeling of the profile shift with depth^19^ in RadCalc. If the dose reference point is within low gradient area, the impact on the dose calculation is minimal (Figure 7, top row). However, in highly modulated beams, such as posterior oblique beams modulated to achieve rectum sparing, if the dose reference point is located in the region of a strong gradient, this may lead to a significant mis‐estimation of the point doses (Figure 7, middle and bottom rows). Overall, these limitations not only affect interpretation of commissioning results, but also potentially impede the ability to establish correlations between MU verification and measurement‐based patient‐specific QA results. Similarly to our results, Sung et al.^6^ reported 2.1 ± 0.8% difference between results of measurement‐based and calculation‐based patient specific QA.

Agreement between RadCalc and the TPS point dose calculation has been evaluated in a set of test cases of increasing complexity. For 39 clinical commissioning plans the mean total point dose discrepancy was −0.3 ± 0.8% (−2.0% to 1.1%) and individual field discrepancies ranged from −15.3% to 6.9%. Upon review, for the one field failing the AAPM TG‐219 consensus action levels,^7^ the dose reference point was located under the leaves for all segments.

### 4.3 Clinical performance

When applied to 4085 clinical plans verified since the last RadCalc software upgrade, the agreement obtained with the model v4 was 0.3 ± 1.1% (−4.8% to 4.2%) opening the way for a reduction of tolerance and action levels based on statistical process control methods.^20^ The number of out‐of‐tolerance results among these calculations amounted to 3 total point dose fails (0.1%) and 13 individual field fails (0.3%).

For comparison, during the first 9 months of operation, for 529 plans, the average agreement was −1.3 ± 1.4% (−6.6% to 5.1%), with 22 total dose fails (4.2%) and 37 individual field fails (7.0%). Most fails (18/22) occurred for oligometastatic treatment plans characterized by smaller and more off‐axis fields compared to other indications. While this data is not presented in the current manuscript, as it was recorded using RadCalc 6.4, not modeling magnetic field effects, this cohort of patients is partially presented in de Leon et al.^18^

After upgrading to RadCalc 7.1, for 2108 plans verified using model v2, the average agreement was 0.6 ± 1.5% (−4.3% to 7.7%), with 38 total dose fails (1.8%) and 35 individual field fails (1.7%). Following the upgrade to RadCalc 7.2, for 4085 plans verified using model v3, the average agreement was 1.1 ± 1.1% (−4.1% to 5.5%), with 15 total dose fails (0.4%) and 28 individual field fails (0.7%).

The performance of the optimized model v4 is comparable to the results presented by Price et al.^5^ who reported 0.1 ± 2.6% agreement for 25 plans on a MRIdian system. Sung et al.^6^ also reported clinical plan results, however, for several different models and divided by indication: prostate −0.9 ± 1.0% to 0.0 ± 1.0%, liver 0.9 ± 0.9% to 1.4 ± 1.0%, and breast 3.5 ± 1.2% to 4.5 ± 1.3%. Higher discrepancies for liver and breast cases may be attributable to the excessive off‐axis ratios described in our work, albeit verifying this would require further insight into their cohort of plans.

### 4.4 Clinical experience and physics light workflow

RadCalc has been in use at our institution for independent MU verification of offline and online adapted treatment plans for the Unity MRL since June 2020.

In the early stages of our clinical MRL program development, independent MU verification was conducted by a physicist present at the treatment console. As the program matured, our center transitioned to a radiation therapist‐led treatment workflow.^21^ Radiation therapists export the reference and the online‐adapted plans for MU verification. Using RadCalcAIR (Automated Import & Report), the plan is automatically imported, calculated, and a PDF report is generated and exported for review and import into R&V system. In the event of a failed MU verification, a notification is automatically triggered. In the intermediate stage of our workflow development, this would enlist the support of physics to investigate. The improved calculation agreement with the TPS obtained in this work has reduced the number of false fails, thereby decreasing the frequency of time‐critical fail investigations. This increased confidence in the QA process has enabled a physics‐light treatment workflow. Most out‐of‐tolerance results occur due to user‐related factors, such as non‐compliance with the plan export procedure or with dose reference point positioning recommendations (e.g., between targets in multi‐target treatments). The remaining small number of genuine fails usually can be attributed to correct but suboptimal dose reference point positioning (i.e., within a dose gradient or under MLC leaves) and can be resolved by adjusting the point location. Currently, those common troubleshooting steps are performed by radiation therapists, with physicists intervening only when required.

By improving model accuracy and automating the MU verification process, we have minimized unnecessary interruptions and reduced the need for immediate physics interventions. This has allowed both physicists and radiation therapists to focus on other aspects of time‐sensitive online planning and treatment, ultimately improving workflow efficiency and the experience for our patients.

## 5 CONCLUSION

This work describes a comprehensive optimization of beam model parameters for a high‐field MRL in RadCalc 7.2, highlighting inherent limitations, such as the absence of cryostat transmission modeling and reliance on semi‐heuristic dose‐to‐water/dose‐to‐medium conversions, while also identifying previously unreported issues, such as suboptimal results for off‐axis calculations and challenges with inhomogeneity corrections. By addressing these limitations and leveraging insights gained from years of clinical use and several software upgrades, we inform the users and suggest areas for future improvements in this widely adopted software.

Our findings are supported by rigorous model commissioning and validation results that met stringent tolerances. The clinical results from a substantial cohort of treatment plans further reinforce confidence in the model and its capacity to support clinicians’ decision‐making.

Achieving high agreement with the TPS has been essential for automating MU verification and facilitating a streamlined, physics‐light treatment workflow. This approach not only supports efficient clinical practice but also ensures high‐quality, patient‐centered care.

## AUTHOR CONTRIBUTIONS

Urszula Jelen, Jack Aylward, Zoë Moutrie, and Michael Jameson designed the research methodology. Urszula Jelen and Jack Aylward conducted data collection. Urszula Jelen analyzed data and drafted the manuscript. Michael Jameson supervised the research. All authors contributed to data interpretation, provided critical revisions, and read and approved the final manuscript.

## CONFLICT OF INTEREST STATEMENT

GenesisCare has a strategic research partnership with Elekta, which includes financial support for MR‐Linac research. Michael Jameson reports speaker honoraria from Elekta AB and a licensing agreement with Standard Imaging Inc.
